# Potential Convergence to Accommodate Pathogenicity Determinants and Antibiotic Resistance Revealed in *Salmonella* Mbandaka

**DOI:** 10.3390/microorganisms12081667

**Published:** 2024-08-13

**Authors:** Na Lv, Jinjing Ni, Shiqi Fang, Yue Liu, Shuang Wan, Chao Sun, Jun Li, Aiping Zhou

**Affiliations:** 1 Jiangxi Key Laboratory of Natural Product and Functional Food, College of Food Science and Engineering, Jiangxi Agricultural University, Nanchang 330045, China; lvna22222@163.com (N.L.); fangshiqi2024@163.com (S.F.); wanshuang999@163.com (S.W.); jackssunc@jxau.edu.cn (C.S.); 2 Department of Laboratory Medicine, Shanghai East Hospital, School of Medicine, Tongji University, Shanghai 200123, China; 3 Laboratory of Bacterial Pathogenesis, Shanghai Institute of Immunology, Shanghai Jiao Tong University School of Medicine, Shanghai 200025, China; nijinjing45@163.com; 4 Department of Microbiology, Shanghai Municipal Center for Disease Control and Prevention, Shanghai 200336, China; liuyue@scdc.sh.cn

**Keywords:** *Salmonella* Mbandaka, hydropericardium, antibiotic resistance and virulence factors, comparative genomics, horizontal gene transfer

## Abstract

*Salmonella* species are causal pathogens instrumental in human food-borne diseases. The pandemic survey related to multidrug resistant (MDR) *Salmonella* genomics enables the prevention and control of their dissemination. Currently, serotype Mbandaka is notorious as a multiple host-adapted non-typhoid *Salmonella*. However, its epidemic and MDR properties are still obscure, especially its genetic determinants accounting for virulence and MD resistance. Here, we aim to characterize the genetic features of a strain SMEH pertaining to *Salmonella* Mbandaka (*S.* Mbandaka), isolated from the patient’s hydropericardium, using cell infections, a mouse model, antibiotic susceptibility test and comparative genomics. The antibiotic susceptibility testing showed that it could tolerate four antibiotics, including chloramphenicol, tetracycline, fisiopen and doxycycline by Kirby–Bauer (K-B) testing interpreted according to the Clinical and Laboratory Standards Institute (CLSI). Both the reproducibility in RAW 264.7 macrophages and invasion ability to infect HeLa cells with strain SMEH were higher than those of *S.* Typhimurium strain 14028S. In contrast, its attenuated virulence was determined in the survival assay using a mouse model. As a result, the candidate genetic determinants responsible for antimicrobial resistance, colonization/adaptability and their transferability were comparatively investigated, such as bacterial secretion systems and pathogenicity islands (SPI-1, SPI-2 and SPI-6). Moreover, collective efforts were made to reveal a potential role of the plasmid architectures in *S.* Mbandaka as the genetic reservoir to transfer or accommodate drug-resistance genes. Our findings highlight the essentiality of antibiotic resistance and risk assessment in *S.* Mbandaka. In addition, genomic surveillance is an efficient method to detect pathogens and monitor drug resistance. The genetic determinants accounting for virulence and antimicrobial resistance underscore the increasing clinical challenge of emerging MDR Mbandaka isolates, and provide insights into their prevention and treatment.

## 1. Introduction

*Salmonella* is a well-known zoonotic pathogen and can be classified into a variety of species, subspecies (classification based on specific fermentability of sugars), and serological variants related to host adaptability, pathogenicity profiles, and antigenic properties [1]. In addition, *Salmonella enterica* is one of the most common pathogens related to foodborne disease outbreaks. According to the statistics released by the Centers for Disease Control and Prevention (CDC) in the United States, infection by *S. enterica* could result in at least one million cases of foodborne diseases each year [2]. A variety of animals, including livestock and poultry, are intermediate reservoirs between *Salmonella* and humans. Most immunocompromised patients infected by *Salmonella* are clinically identified along with gastroenteritis. Remarkably, *Salmonella* may spread from the gut to the blood [3]. Cardiovascular symptoms occur in nearly 1–5% of patients suffering *Salmonella* infections [4], likely related to the adhesion capability of *Salmonella*, the burden of bacteria and the host immune responses [5].

Currently, serial antibiotics have become an important therapy for the treatment of *Salmonella* infection. Meanwhile, antimicrobial drugs have also been heavily adopted by livestock and poultry production. Nearly 73% of the antibiotics worldwide are used to raise animals [6]. However, the increasing emergence of multidrug resistant (MDR) bacteria is commonly attributed to the abuse of antibiotics, greatly challenging the prevention and control of foodborne diseases [7,8].

*S. enterica* serovar Mbandaka (*S.* Mbandaka) is non-typhoid and can infect multiple hosts such as bovines, poultry and humans. Serovar Mbandaka was first isolated and typed in Belgian Congo in 1948. Currently, a worldwide spread of Mbandaka has been observed due to its relatedness with meat and cereal consumption, posing a major threat to food safety (a cause of foodborne diseases) and human health (such as endovascular complications) [9,10,11]. In the EU/EEA, Israel and the UK, a multi-country outbreak of *S*. Mbandaka is likely correlated with the consumption of chicken meat [12]. The European Union has listed it as one of the top ten serotypes responsible for outbreak cases of human salmonellosis [13]. As well, foodborne disease related to this serotype has been detected in several states of the USA, involving ready-to-eat breakfast cereal [9]. In addition, MDR *S.* Mbandaka isolates are increasingly determined, involving aminoglycosides, tetracycline and sulfonamides. Of note, an MDR strain classified as *S.* Mbandaka was isolated from a patient with mitral valve endocarditis and having difficulty with anti-infection treatment, likely resulting from cereal consumption during a Mbandaka outbreak [3]. However, insufficient epidemics surveys on Mbandaka have resulted in inefficiency in its prevention and control outbreak, although serum agglutination or sequencing technologies benefit its convenient diagnosis [14]. Nevertheless, these cases imply that this serotype spreads by diverse routes and its distinguishing infection patterns. However, the genetic determinants accounting for its antibiotic resistance and infection ability are still obscure [8,10].

Furthermore, the number of fully sequenced strains pertaining to *S.* Mbandaka is limited. In this scenario, more isolates and their genomes are essential to further understand the epidemiological and phylogenetic characteristics of serovar Mbandaka. In this study, we isolated a strain pertaining to *S.* Mbandaka from the hydropericardium of a critically ill patient. We attempted (1) to examine its antimicrobial resistance (AMR) and its virulence heterogenicity; and (2) to investigate its genetic determinants correlated with pathogenicity and drug resistance. Therefore, our findings could facilitate insights into the genetic diversity of the virulence factors and drug resistance in *S.* Mbandaka.

## 2. Materials and Methods

### 2.1. Bacteria Isolation and Culture

Strain SMEH was isolated from a patient’s hydropericardium in Shanghai, China in 2021. A 5 mL pericardial fluid sample was inoculated onto Biphase Blood Culture (Thermo Scientific, Waltham, MA, USA). If the blood culture was positive, 0.5 mL cultures were transferred to blood agar plate and MacConkey agar. The purified strain was acquired from the resulting blood culture and the colony on MacConkey/Xylose Lysine Deoxycholate (XLD) agar indicated the occurrence of *Salmonella*. Next, single colonies from pure cultures were characterized using a VITEK 2 COMPACT automated system (bioMérieux, Lyon, France) [15] and confirmed by matrix-assisted laser desorption/ionization time-of-flight mass spectrometry (MALDI-TOF MS, Zybio, Chongqing, China) [16].

Luria Bertani (LB) broth: 5 g/L NaCl, 5 g/L yeast extract, and 10 g/L tryptone. 2% agar was added to prepare LBA (for plate counting, Sangon Biotech, Shanghai, China). Mueller–Hinton agar (MHA, for testing antibiotic susceptibility). Blood agar plates and selective medium—MacConkey agar were commercially available (Thermo Fisher, Waltham, MA, USA); XLD agar was purchased from CHROmagar Microbiology (Paris, France). Bacterial culture of 18–24 h was conducted in an incubator at 35 ± 1 °C (supplemented with 5% CO_2_ if blood culture was conducted).

### 2.2. Serotyping

The isolate identified as *Salmonella* spp. was subjected to serotyping, according to the Kaufmann–White classification using slide agglutination with O and H antigen-specific anti-sera (Bio-Rad, Marnes-La Coquette, France; Staten Serum Institute, Copenhagen, Denmark; Sifin, Berlin, Germany) at the Shanghai Municipal Center for Disease Control and Prevention (Supplemental Table S1).

### 2.3. Antibiotic Susceptibility Testing

Antimicrobial susceptibility testing of SMEH isolates was performed using VITEK 2 Compact AST-N335 broth micro-dilution technology (bioMérieux, France), Kirby–Bauer (K-B) strip on MHA plates (Oxoid, Wesel, Germany). A total of thirty-one antimicrobials (26 for broth microdilution and 17 for disc diffusion, respectively) were applicable to this susceptibility testing (Table 1, purchased from Oxoid, Wesel, Germany). Minimum inhibitory concentrations (MICs) were interpreted according to breakpoints recommended by Clinical and Laboratory Standards Institute (CLSI) [17]. The inhibition zone was determined by K-B disc diffusion assays. Briefly, antibacterial activity was evaluated by measuring the diameter of circular inhibition zones. The un-inoculated medium was also prepared as negative control, while *Escherichia coli* ATCC 25922 (ATCC: American Type Culture Collection) was selected as positive control. The microbial suspension was properly diluted to 0.5 McFarland turbidity for subsequent VITEK 2 Compact and K-B testing. In brief, a mixture solution was prepared with 3 mL 0.45% saline and 145 μL suspension for VITEK 2 Compact [15]. And bacterial suspension of the appropriate volume was plated on MHA, and then K-B strips were transferred onto MHA [18]. Of note, both broth micro-dilution and K-B tests were performed in triplicate. The testing outputs were regarded to be accurate, if MIC values and the inhibition zones of ATCC 25922 were acceptable in the range of quality control. As recommended, pan drug resistance (PDR) indicates resistance to all antibiotics, extensive drug resistance (XDR) denotes susceptibility to 1–2 types of antibiotics and resistance to other antimicrobials, and multidrug resistance represents acquired resistance to ≥3 types of antibiotics (excluding intrinsic resistance) [7].

### 2.4. Cell Infection Assays

Murine macrophage-like cell line RAW 264.7 were employed to determine the intracellular replication capability of *Salmonella*. HeLa cells were employed to check the invasion rate of epithelial cells [19,20,21]. RAW 264.7 and HeLa cells were obtained from the Cell Resource Center of the Shanghai Academy of Sciences (Shanghai, China). Briefly, 2 × 10^5^ RAW 264.7 cells were seeded into each well of 24-well plates in Dulbecco modified Eagle medium (DMEM, Thermo Fisher, Waltham, MA, USA) supplemented with 10% fetal bovine serum (FBS, Thermo Fisher, Waltham, MA, USA). Cells were infected at multiplicity of infection (MOI) of 10 with strains 14028S and SMEH. After incubation for 1 h at 37 °C, extracellular bacteria were killed by 100 mg/mL gentamicin for 2 h. Then the mixture was switched to medium containing 25 mg/mL gentamicin for the remainder of the experiment. Infected cells were lysed with phosphate buffered saline (PBS, pH 7.2 ± 0.2) containing 0.1% Triton X-100 and plated on LB agar after dilution. The fold change in bacterial replication from 2 h to 24 h post-infection in cells was measured by colony forming unit (CFU) counting. Likewise, HeLa cells were seeded at 1 × 10^5^ cells per well in 24-well plates in DMEM with 10% FBS and infected at MOI of 100 with four strains. After 1 h of infection, cells were washed twice with PBS and incubated in DMEM-10% FBS plus 100 mg/mL gentamicin for 2 h to kill extracellular bacteria. Then cells were washed twice with PBS and lysed as described above. Lysates were plated on LB agar and counted to calculate the invasion rate. Each independent assay was performed simultaneously in two separate wells and repeated three times.

### 2.5. Animal Experiments

All animal procedures were approved by Shanghai Jiao Tong University School of Medicine, and this study was carried out in strict accordance with the National Research Council Guide for Care and Use of Laboratory Animals [SYXK (Shanghai 2007-0025)]. Animal experiments were performed as described previously [19]. Inbred 8-week-old C57 mice were deprived of food and water for 4 h prior to administration of 20 mg of streptomycin per mouse by oral gavage. After 4 h, food and water were provided *ad libitum*. After 20 h streptomycin treatment, food and water were withdrawn again for 4 h. Afterward, 1.5 × 10^7^ CFU bacteria in 200 μL PBS was administered by oral gavage, and control mice were given 200 μL PBS. Water and food were provided *ad libitum*. The five mice from each group were left until 15 d post-infection when the experiment was terminated. To record survival rate, the number of live mice were counted twice daily.

### 2.6. Genomic DNA Extraction and Sequencing

Genomic DNA was extracted using Gentra Puregene Yeast/Bact. Kit (Qiagen, Valencia, CA). Illumina HiSeq X10 platform (Illumina Inc., San Diego, CA, USA) and GridION X5 platform (Oxford Nanopore, Oxford, UK) was used for sequencing SMEH at the Zhejiang Tianke (Hangzhou, China). After filter processing of raw sequencing reads (the paired-end short illumina reads and the long nanopore reads), the genome assembly was performed using Unicycler (v0.4.5) [22]. The resulting genome includes a circular chromosome and three circular plasmids, further annotated according to the Prokaryotic Genome Annotation Pipeline (PGAP, www.ncbi.nlm.nih.gov/genome/annotation_prok (accessed on 14 June 2022)).

### 2.7. Bioinformatics

Annotation of assembled draft genomes was conducted by Prokka v1.12 [23]. Replication origins were assessed by Ori-Finder [24]. In addition, protein function was manually annotated by comparing protein sequences with the Non-Redundant Protein Sequence Database (NR, ftp.ncbi.nih.gov/blast/db), Swiss-Prot (www.ebi.ac.uk/uniprot), KEGG (www.genome.jp/kegg) and COG (www.ncbi.nlm.nih.gov/COG) databases. CVTree3 (available at tlife.fudan.edu.cn/cvtree) was used to plot a whole-genome-based phylogenetic tree. This phylogeny was visualized using Interactive Tree of Life (iTOL) (available at itol.embl.de). Subcellular localization prediction was performed using PSORTb (www.psort.org) based on the signal peptides in the protein sequence. The potential prophage was searched with PHASTER (phaster.ca). Genomic islands were predicted using the IslandViewer4 (www.pathogenomics.sfu.ca/islandviewer). BLASTp-derived programs were used to compare the amino acid sequences of strain SMEH with VFDB (www.mgc.ac.cn/VFs)/CARD (card.mcmaster.ca)/ISfinder (isfinder.biotoul.fr) database (E-value ≤ 1 × 10^−5^), to respectively collate virulence factors, drug resistance genes, and insertion sequences. ResFinder 4.0 (cge.cbs.dtu.dk/services/ResFinder) was also used to analyze antibiotic resistance genes and chromosomal mutations that mediate microbial resistance. Horizontal genetic elements including integrative conjugative elements (ICEs) and their accessory modules were predicted using VRprofile [25]. Type VI secretion system (T6SS) was identified by SecReT6 [26]. BLAST Ring Image Generator (BRIG)-based assays were conducted to generate circular comparison for *Salmonella* genomes [27]. Unless specially stated, these databases and analyses related to comparative genomics were accessed on 14 June 2022.

## 3. Results

### 3.1. Clinical History

**Figure 1:**
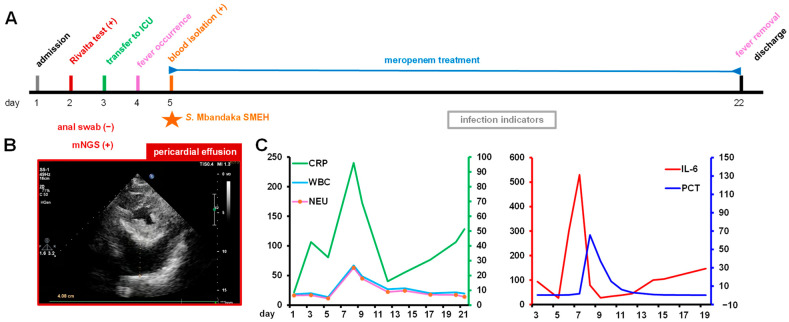
Clinical information of the 83-year aged patient. (**A**) Clinical history. ★ Blood isolation for *S.* Mbandaka strain SMEH. mNGS: metagenomic next-generation sequencing (**B**) B-scan ultrasonography. (**C**) Infection indicators. A series of infection indicators were measured during the prolonged antibiotic treatment. Of note, CRP: C-reactive protein (mg/L), WBC: white blood cell (10^9^/L), NEU: neutrophil (10^9^/L), IL-6: interleukin-6 (pg/mL), PCT: procalcitonin (ng/mL).

On 25 August (recorded as day 1), this patient (information was anonymized, exemption from informed consent) was admitted to the hospital, with a positive Rivalta test and pericardial effusion (determined by B-scan ultrasonography). In addition, metagenomic next-generation sequencing (mNGS) indicated a positive risk for *Salmonella* infection (by mapping sequencing reads against NR). This patient was therefore transferred to the intensive care unit (ICU) alongside an occurrence of relapsing fever (Figure 1A,B).

It was noteworthy that the *S.* Mbandaka strain SMEH was isolated from the blood specimen on day 5. From day 5 to 21, this patient suffered anti-infection treatment using meropenem, and the infection indicators (CRP, WBC, NEU, IL-6, and PCT) were measured (Figure 1C). On day 22, this aged patient was eventually discharged with no remaining pericardial effusion and an obvious condition improvement. This finding suggested that *S.* Mbandaka could be the likely causative agent of pericardial effusion and concurrent infections.

### 3.2. Isolation and Characterization

The candidate isolate was selectively picked from the XLD agar plates, and purified colonies were finally acquired (hereafter, termed as SMEH, Supplemental Figure S1). The morphology of the colony can be described as a clasp-like colony, with a large glossy black center surrounded by the mucous margin. In brief, the colony morphology was in accordance to typical *Salmonella*.

We then retrieved five genomes (FORC_015, ATCC 51958, GJ0703, CT18 and 14028S) archived in the National Center for Biotechnology Information (NCBI), of which the serotypes were, respectively, appointed as Typhimurium, Mbandaka, Rissen and Typhi (the serotyping is shown in Supplemental Table S1). To further confirm the serotype of strain SMEH, anti-sera were applicable to typing O and H antigens by slide agglutination. The O and H1,2 antigen of strain SMEH were determined to be 7, z10, e,n,z15, consistent with representative Mbandaka. As a result, we therefore classified this strain into *Salmonella* Mbandaka by comparing immunization schedules of O and H antigens.

### 3.3. S. Mbandaka SMEH Is Resistant to Four Antibiotics

We examined the antibiotic susceptibility of strain SMEH by determining the MICs and inhibition zones against 26 and 17 drugs, respectively. The MICs and susceptibility interpretations of the above-described strain SMEH are shown in Table 1 (no clinical breakpoints for streptomycin, colistin and azithromycin). Antimicrobial susceptibility testing experiments showed that the SMEH strain was resistant to at least four antibiotics, including chloramphenicol, tetracycline, fisiopen, and doxycycline. In addition, SMEH moderately or intermediately tolerated three antibiotics including ciprofloxacin, levofloxacin and minocycline, and was highly sensitive to the remaining antibiotics. Collectively, SMEH could be classified as an MDR *S.* Mbandaka (insusceptible to ≥3 types of antibiotics).

### 3.4. S. Mbandaka SMEH Promotes Intracellular Survival and Invasion

**Figure 2:**
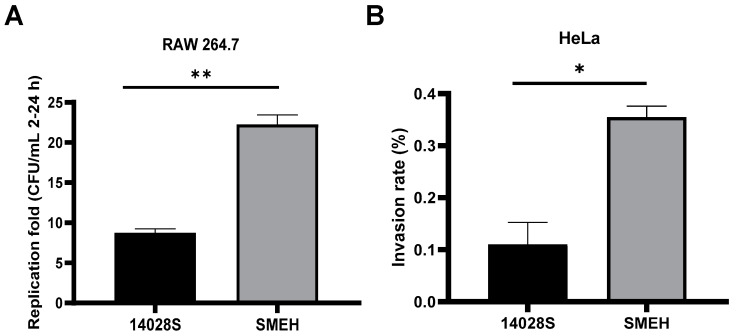
Cell effects of infection with *S.* Mbandaka strain SMEH. (**A**) The proliferation capability in RAW 264.7 macrophage cells. At 2 or 24 h post-infection, bacterial colonies were counted by plating an aliquot of cell lysates on LB agar. (**B**) The invasion efficiency in HeLa cells. At 1 h post-infection, bacterial colonies were counted by plating an aliquot of cell lysates on LB agar. Results are shown as mean ± standard deviation. Statistical significance was determined with the unpaired Student’s *t*-test using GraphPad Prism 8.0 (GraphPad Software, San Diego, CA, USA). *: *p* < 0.05, **: *p* < 0.01.

To determine the intracellular survival of SMEH, we infected the mouse macrophage-like RAW 264.7 cells with *S.* Typhimurium 14028S and *S.* Mbandaka SMEH. From 2 h to 24 h post-infection, intracellular bacterial counting showed that SMEH replicated by ~22 folds, and 14028S had an approximately 9-fold replication rate (Figure 2A). Next, we performed the invasion assay in HeLa cells to measure infection capability of strain SMEH and 14028S. Likewise, SMEH showed a ~3-fold invasion rate compared to 14028S (Figure 2B). Altogether, these data showed that *S.* Mbandaka SMEH has augmented invasiveness and reproducibility compared to *S.* Typhimurium 14028S.

### 3.5. S. Mbandaka SMEH Is Less Virulent Than S. Typhimurium 14028S in Mouse Model

**Figure 3:**
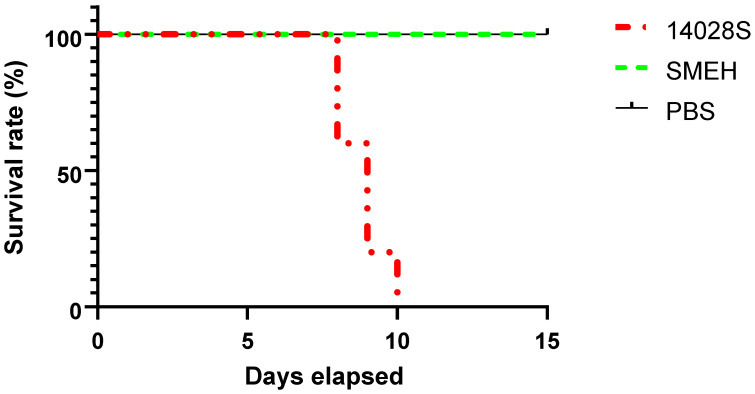
Comparative survival analysis between strains SMEH and 14028S in mouse model. The mortality of mice was recorded twice per day. Mantel–Cox test was performed between SMEH and 14028S-infected mice. PBS: control group inoculated with phosphate buffered saline.

To further evaluate the virulence of *S.* Mbandaka strain SMEH, mice were inoculated orally with 1.5 × 10^7^ CFU of *S.* Mbandaka strain SMEH or *S.* Typhimurium strain 14028S, and mortality was monitored twice per day. Mice in the PBS group were alive and healthy throughout the study. Mice infected with strain 14028S began dying after 8 d post-infection and all died by 10 d post-infection (Figure 3). However, mice in the SMEH strain group showed slight infection-related morbidity, such as ruffling of fur and wasting at 2 d post-infection but recovery after 3 days. Interestingly, all mice infected with strain SMEH were alive for the whole experiment period, indicating that *S.* Mbandaka shows less virulence compared with *S.* Typhimurium in the mouse model. This finding prompted us to reveal the genetics determinants that are likely related to virulence factors, in order to find a reason for the contradictory phenomena found in the mouse model and cell assay.

### 3.6. Conserved and Specific Genes Compared to Salmonella Representatives

**Figure 4:**
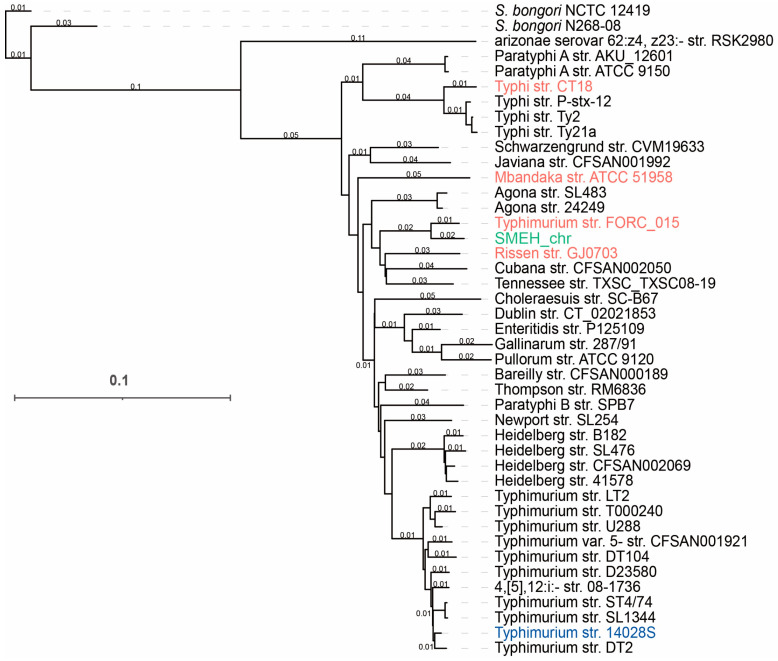
Phylogeny of strain SMEH and other representatives pertaining to different serotypes of *Salmonella.* SMEH and other *S.* Mbandaka constitute a compact group in this tree. Phylogenetic relationships were inferred by CVTree3 (available at tlife.fudan.edu.cn/cvtree (accessed on 14 June 2022)), and evolutionary distances were denoted adjacent to the phylogenetic clades. Note: SMEH_chr indicates strain SMEH chromosome. 14028S (virulence comparison) was colored by blue, while other representative serotypes found in *Salmonella* strains (Supplemental Table S1) were indicated by red.

**Figure 5:**
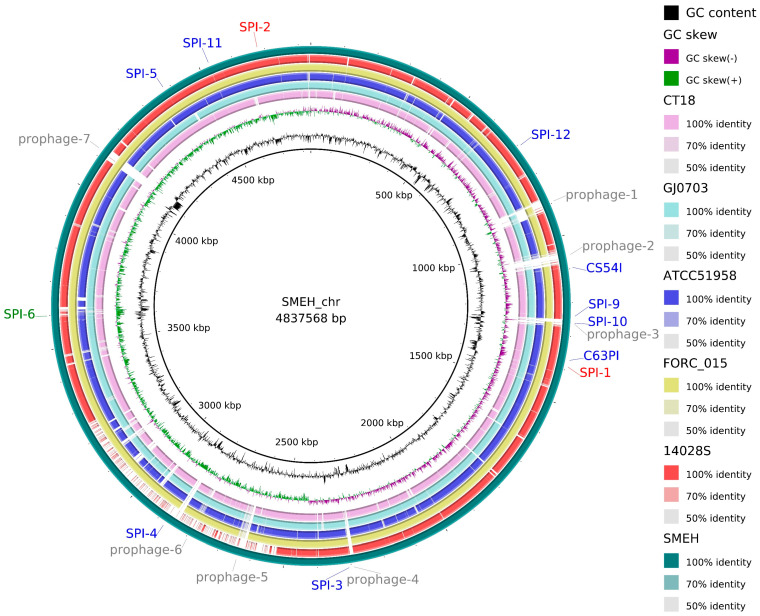
Circular schematics for strain SMEH chromosome. From inner to outer circles: GC content, GC skew, homologs in strain CT18, homologs in strain GJ0703, homologs in strain FORC_015, homologs in strain ATCC 51958, homologs in strain 14028S, and mobile genetic elements identified using VRprofile (bioinfo-mml.sjtu.edu.cn/VRprofile (accessed on 14 June 2022)). Customized CGview (https://proksee.ca (accessed on 15 January 2023)) and VRprofile analyses were performed to generate circular comparisons. The predicted *Salmonella* pathogenicity islands (SPIs) and other virulence determinants were indicated and drawn to scale, respectively. Note: SMEH_chr indicates strain SMEH chromosome.

The strain SMEH chromosome length was 4.84 Mb, and G + C content was 52.11%. Moreover, three plasmid replicons were acquired and assembled, whose sizes were 10,047 bp, 9208 bp, and 7769 bp. Their G + C% were 50.58%, 55.98%, and 62.14%, respectively. Then we annotated 4743 genes, including 118 RNA genes and 4511 ORFs (open reading frames) in the genome of strain SMEH. A total of 42 representative *Salmonella* with fully sequenced genomes were collected from the NCBI database. Subsequently, a whole-genome-based phylogenetic tree was generated using CVTree3 [28]. We found that the phylogenetic tree clade of strain SMEH is closer to the counterpart of strain FORC_015 than those in the other three representative strains (Figure 4). Of note, the clan of strain 14028S is distantly related to that of strain SMEH. General genomic information of six strains (CT18, FORC_015, ATCC 51958, 14028S, GJ0703 and SMEH) was compared (Figure 5 and Supplemental Table S2) to show their heterogeneity and genetic variants, including *Salmonella* pathogenicity islands (SPIs) and secretion systems. For example, SPI-1, SPI-2 and SPI-6 homologs related to infection, survival and replication ability were identified in strain SMEH.

This diversity prompted us to determine their conserved and specific genes. We thus plotted the circos-based comparison for strains 14028S, CT18, GJ0703, FORC_015, ATCC 51958, and SMEH. A variety of genetic acquisitions or losses were observed between strains SMEH and 14028S (Supplemental Figure S2A). 463 and 1225 specific genes were identified in strain SMEH and 14028S, likely responding for their virulence differences in cell infection and the mouse model (Supplemental Figure S2B). The corresponding results showed that the sequence similarity between strain SMEH and strain FORC_015 was higher than that between strain SMEH and ATCC 51958 (Supplemental Figure S3A). Moreover, both gene re-organization and loss/acquisition were observed by comparing SMEH and ATCC 51958 genomes. The gene organization between genomes SMEH and FORC_015 was more co-linear than that between strains SMEH and ATCC 51958. Nevertheless, we further confirmed that the serotype of isolate SMEH was Mbandaka.

In addition, the core genes, and specific genes in the SMEH chromosome were identified based on the BLASTp-and COG alignment. Pan-genomics driven by all-against-all BLASTp indicated that the SMEH genome was similar to FORC_015 and ATCC 51958 rather than CT18 and GJ0703 (Supplemental Figure S3B). The COG classification (as of June, 2022) showed that the five strains were substantively different in the genes accounting for energy production and conversion (C), amino acid transport and metabolism (E), carbohydrate transport and metabolism (G), transcription (K), replication, recombination, and repair (L) and intracellular trafficking, secretion, and vesicular transport (U). A total of 550 genes with unknown function (S) and 70 defense-related genes (V) were predicted in the strain SMEH chromosome (Supplemental Figure S3C). SMEH has 221 genes associated with L, but only 193 candidates in FORC_015. Additionally, 168 genes related to U were found in CT18, while 205 genes were assessed to be related to U in SMEH. Together, our findings indicate that strain SMEH is closely related to strains FORC_015 and ATCC 51958, alongside a variety of variants among their genomes.

### 3.7. Genetic Diversity of MLST and Serotype

Noteworthy, the typing methods of genus *Salmonella* usually include phenotyping and genotyping. Physiological and biochemical testing is a key procedure in microbial characterization, with great significance for epidemiologic investigation, disease diagnosis and public health. In addition, antigen serotyping and multi-locus sequence typing (MLST) are the most common methods for characteristic and molecular typing, respectively [29]. To further define the serotype of strain SMEH, we also typed the MLST for these *Salmonella* strains (as shown in Supplemental Table S2). The MLST results showed that both strain SMEH and FORC_015 were ST-413, and the generic serotype of ST-413 *Salmonella* archived in the EnteroBase database (enterobase.warwick.ac.uk (accessed on 15 January 2023)) was Mbandaka. Interestingly, the serotype of strain FORC_015 was previously considered as Typhimurium (Supplemental Table S1), greatly suggesting a divergence for the serotypes of *Salmonella* strains pertaining to the same MLST.

**Figure 6:**
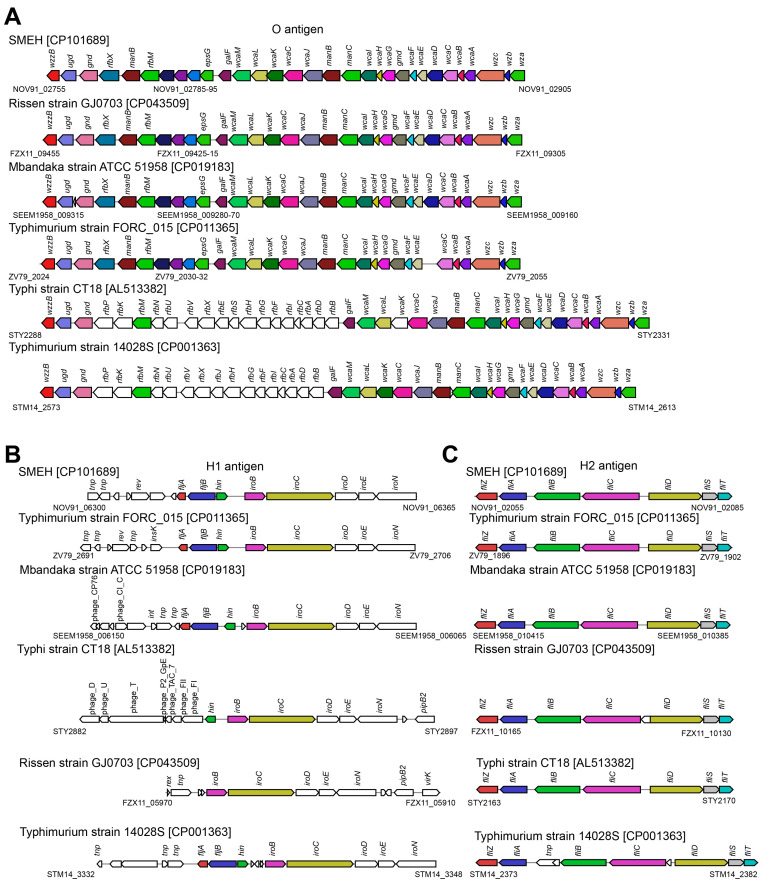
The co-linearity of gene clusters responsible for O- (**A**) and H1,2- (**B**,**C**) antigen biosynthesis amongst strains SMEH, CT18, FORC_015, GJ0703, ATCC 51958 and 14028S. These comparative schematics were calculated by multigeneblast (multigeneblast.sourceforge.net (accessed on 14 June 2022)) and drawn to scale.

The genetic variants associated with O- and H-1,2 antigen synthesis were compared to explain this discrepancy. A significant loss of *rfb* genes within the O-antigen gene clusters was observed for strain SMEH compared to CT18 (Figure 6A). Moreover, serial properties (prophage or transposase genes) related to horizontal transfer were determined around *fij* operons accounting for H1-antigen biosynthesis, whereas a good synteny in *fli* genes for H2-antigen (Figure 6B,C). Notably, we predicted *gtrA* as the bio-signature of SPI-16, although the intact SPI-16 was not found [30]. NOV91_04275 and NOV91_05305 are orthologues of GtrA, with 81.7% and 83.3% identity to progenitor in strain CT18, respectively (Supplemental Table S3). And *gtrA* could play a role in O-antigen glucosylation to convert the serotype [31]. Interestingly, two *gtrAB* operons (*NOV91_04275-80* and *NOV91_05305-10*) are located within prophage-1 and -2, respectively (Figure 5). As a result, we assessed that these two mobile elements were seroconverting bacteriophages, benefiting long-term colonization.

### 3.8. Salmonella Pathogenicity Islands

The pathogenicity and drug resistance risk of the strain SMEH was determined by comparative genomics and handy curation. Both single gene and gene cluster scale were investigated by VRprofile, VFDB, IslandViewer and CARD, including potential SPIs and AMR determinants. In brief, 11 *Salmonella* pathogenic islands (C63PI, CS54I, SPI-1 to 6, SPI-9, SPI-11, SPI-12) were detected. Of note, two type III secretion systems (T3SSs) were located on SPI-1 and SPI-2, respectively (Supplemental Table S3). The invasion and replication ability of *Salmonella* have been attributed to these two SPI prototypes, likely explaining the SMEH virulence difference detected in cell assay and mouse model.

In addition, an intact type VI secretion system (T6SS) gene cluster was determined using SecReT6, and a total of six immunity proteins were predicted as antagonists to combat cognate effectors. For example, *NOV91_17370* coding for a candidate Shiga toxin, while adjacent *NOV91_17365* was found to encode the immunity protein belonging to the Tai family (constituting the type VI amidase effector-immunity complex). Likewise, *NOV91_17335* and *NOV91_17340* were identified as Tae4 and Tai4 homologues, respectively. Unprecedentedly, a paired Imm52 and Tox-REase-5 were present in the vicinity of the additional T6SS in strain ATCC 51958. Generally, SPI-6 is a genomic island bearing a T6SS, alongside a mosaic structure coding for S-fimbrial adhesin (*saf*) and/or Typhi colonization factor (*tcf*) operons [32]. The collinearity analysis indicated that the T6SS in strain SMEH was highly identical to those carried by SPI-6 in strains FORC_015, ATCC 51958, TXSC-TXSC08-19, CT18, and GJ0703, although a series of variants and diversity in SPI-6 was identified in strain SMEH genome. It is noteworthy that strain ATCC 51958 (the prototype of serovar Mbandaka) encodes an additional T6SS co-localized with one integrative conjugative element (ICE, ranging from *SEEM1958_022095* to *SEEM1958_022215*) (Supplemental Figure S4). T6SS and ICE have been re-considered as auxiliary factors in monitoring intracellular homeostasis and adaptability. This genomic variant might confer fitness advantages upon serovar Mbandaka if confronting stresses.

### 3.9. A Reservoir to Accommodate AMR Genes in SMEH Plasmids

**Figure 7:**
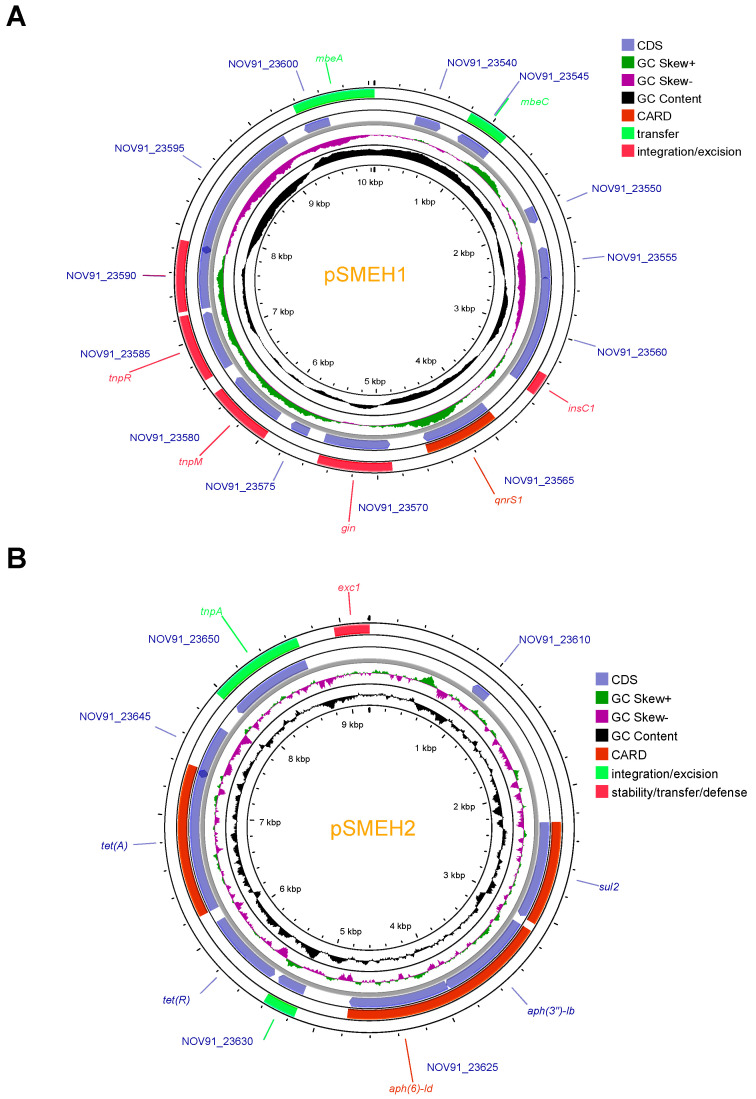

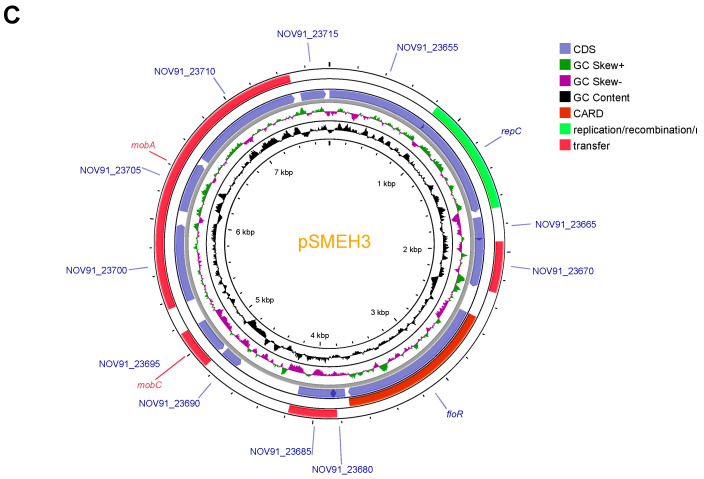
Circular schematics for 3 plasmids (pSMEH1-3, (**A**–**C**)). From inner to outer circles: GC content, GC skew, CDS (coding DNA sequences). Customized CGview and VRprofile analyses were performed to generate circular comparisons. Antibiotic genes were predicted by CARD and ResFinder.

A total of twenty-one AMR-related regions were detected in the chromosome, while six and five candidates were detected in pSMEH2 and pSMEH3, respectively (such as *qnrS* and *sul2, as* shown in Figure 7). Obviously, SMEH is a potential pool to acquire and transfer AMR genes. We found that the genetic architectures of these plasmids of strain SMEH were highly specific compared to those of strains with the same serotype.

**Figure 8:**
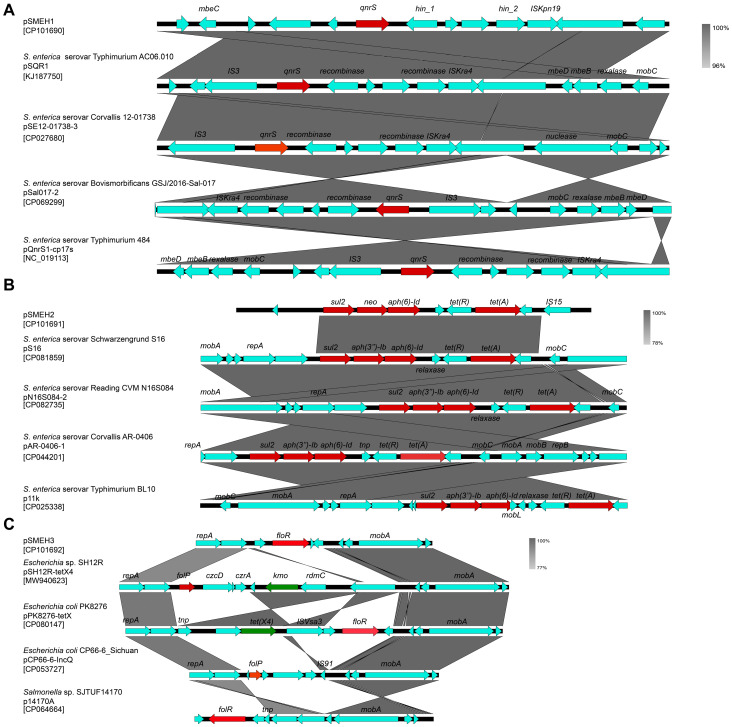
Linear alignment for three plasmids and their homologues. (**A**–**C**) Synteny of pSMEH1-3 and their homologous plasmids were generated by Easyfig (easyfig.sourceforge.net). Homologous regions are marked by gray shading. Notably, *sul2*: sulfanilamide resistance gene; *tet(X4)*: tetracycline-inactivating monooxygenase gene; *folP*: gene encoding dihydropteroate synthase; *floR*: gene encoding chloramphenicol/florfenicol efflux MFS transporter; *tnp*: transposase gene; *neo*: gene responsible for neomycin/kanamycin resistance; *qnrS*: gene coding for Qnr family pentapeptide repeat protein. *tet*(X4) homologues were flagged with green, while other genes related to antibiotic resistance were labelled by red.

Therefore, we assayed the synteny of the SMEH plasmids with their homologs (Figure 8). *qnrS* is a generic resistance determinant of quinolone and was observed on pSMEH1-like plasmids. Comparative genomics analysis of pSMEH2 with its homologs revealed the landscape of the plasmid-bearing genes involved in resistance to aminoglycoside, sulfonamide, and tetracycline antibiotics. Genes co-localized in pSMEH3 mainly include *repA*, *floR*, and *mobA*. In addition, co-linearity unraveled a high similarity between its *repA*-*mobA* pattern and homologs in other plasmids from *E. coli.* Interestingly, a similar architecture of sequences was also observed in plasmid p14170A of *Salmonella* sp. SJTUF14170. It is plausible that pSMEH3 can be considered an intermediate/ancestor for *tet*(X4)-like member (tigecycline-resistance) transferability. In pSMEH3, we also detected *floR* accounting for the chloramphenicol resistance. FloR proteins belong to the major facilitation factor superfamily (MFS superfamily), driven by proton-driving forces transporting sugars, metabolites, anions, and drugs. For this member of the MFS superfamily, drug efflux pumps may facilitate this strain that is highly resistant to chloramphenicol/florfenicol.

The abundance of genes encoding transposase/integrase in the context of these resistance factors suggests that these resistance genes are continuously evolving and spreading. pSMEH1 and pSMEH2 contain insertion elements such as IS*Kpn19* or IS*15*, likely responsible for the transposition or insertion of adjacent genes. In general, these plasmids generally share a similar backbone and key genetic determinants compared to the other homologous plasmids, but they also differ in their gene organization and loss/acquisition of AMR genes (Figure 8). pSMEH2 was deficient for the *mob* genes involved in plasmid transferability and the *rep* genes involved in plasmid replication. This indicates that pSMEH2 cannot replicate and transfer autonomously, perhaps resulting in co-evolution for the pSMEH2 and SMEH chromosome. Moreover, the synteny of pSMEH3 showed the heavy recombination events and its potential to accommodate *tet*(X4), conferring tigecycline resistance from its FAD-dependent monooxygenase activity [33]. Previously, clinical therapy with multiple antibiotics was conducted to treat infections in this patient. However, a series of antibiotics such as tetracycline was determined to be ineffective. Furthermore, we assessed that pSMEH3 may bear an insertion hotspot for *tet*(X4).

## 4. Discussion

*S. enterica* causes self-limiting diarrheal illness, and the elderly and children are prone to complications such as dehydration, bacteremia, and infections outside the gastrointestinal tract, posing a serious threat to human health [9]. Previously, Mbandaka was a relatively uncommon serotype in *Salmonella*. Of note, in the US reports, the majority of Mbandaka strains isolated from poultry were pan-susceptible to a variety of clinical drugs, including third-generation cephalosporins and fluoroquinolones [34]. However, a few strains of this serotype were determined resistant to multiple antibiotics, resulting in a long-term neglect on their genomic surveillance. In addition, *Salmonella* infections are often misidentified before laboratory identification and genomics analysis. A positive isolation was observed in liquid from the pericardium, while no *Salmonella* was found in the anal swab. In this retrospective case, mNGS combined with genomics analysis could benefit from appropriate antimicrobial and effective surveillance.

Overall, strain SMEH was found resistant to four antibiotics: chloramphenicol, tetracycline, fisiopen, and doxycycline and intermediately sensitive to three antimicrobials: ciprofloxacin, levofloxacin, and minocycline. Also, it is noteworthy that the MDR profiles of *S.* Mbandaka have been an emerging public concern, including resistance to tetracycline, sulfonamides, aminoglycosides and fluoroquinolones [10,14]. From this scenario, the emergence of this multidrug-resistant *S.* Mbandaka could challenge therapeutic applicability. Next, we found that the virulence of strain SMEH in mice is significantly lower than that of S. Typhimurium strain 14028S. A total of five mice died from treatment with strain 14028S. Further, all mice survived if strain SMEH was inoculated by gavage administration, although the occurrence of infection symptoms was found, followed by rapid recovery. In contrast, we found that strain SMEH has an obvious ability to replicate in RAW 264.7 macrophages and to invade HeLa cells. Of note, this ability is an important indicator to measure the potential virulence of *Salmonella* [15]. However, more clinical experiments are still required, such as the identification of the ability to reproduce in the human macrophage U937 and an intraperitoneal infection to mimic the systemic phase of infection. Bacterial loads in the liver, spleen, and heart are complemented bypassing the entry of bacteria through epithelial cells into the gut.

To adapt to the different environments, complex immunity responses, and microbiome competition in the host, many virulence and AMR factors were encoded to promote bacterial growth and persistence in the host [35]. We thereby attempted to reveal the genetic variants that confer multiple virulence and antibiotic resistance upon strain SMEH by comparing the genomes of *Salmonella* representatives. As well, conserved and specific genes responsible for serotyping antigen synthesis were determined in this ST-413 strain, such as *gtrA*. Our findings suggested the on-going genetic variants of *S.* Mbandaka, contributing to the dissemination and spread of this serovar.

Numerous virulence genes related to *Salmonella* pathogenicity usually co-localized in SPIs, virulence plasmids and prophages [36]. Among these pathogenicity factors, both fimbrial and nonfimbrial adherence determinants are correlated with bacterial attachment and colonization, greatly contributing to the persistent infection in *Salmonella*. For example, MisL is an autotransporter encoded by SPI-3, identified as an extracellular matrix adhesin involved in intestinal colonization and modulating the intestinal persistence of *S. Typhimurium* in mouse models [37]. In addition, the capacity to uptake iron and magnesium ions also affects persistence during infection. MgtC is a key gene for adapting cellular pathogens to low Mg^2+^, regulating intracellular energy demands and micro-niche pH [15].

SPIs are considered to be acquired from other organisms through horizontal transfer [38]. Most genes on SPIs are related to an adaptive ability to invade, reproduce, and survive within host cells [39]. These effectors rely on bacterial secretion systems to export them into the host cells [40,41]. Two type III secretion systems, namely T3SS-1 and T3SS-2, and their effectors were found on SPI-1 and SPI-2, respectively [38]. T3SS-1 modulates host immune responses, such as immune cell recruitment and apoptosis as well as biofilm formation critical for infection and invasion [42]. And T3SS-2 is critical for survival within host cells and macrophages [43]. C63PI participates in bacterial invasion as the iron transport system in SPI-1 [44]. Of note, T3SS-1 effector genes (*spiABCD*) and T3SS-2 effectors genes (*sseJ* and *sseK2*) were identified in strain SMEH as well, required for a systemic infection in a mouse model [45].

T6SS usually carried by SPI-6 enhances the bacterial ability to invade and infect epithelial cells [46]. Interestingly, the genomes of Dublin serotypes have two T6SS with different gene organization. Both these two T6SSs are localized on *Salmonella* pathogenic islands (T6SS_SPI-6_ and T6SS_SPI-19_), and only T6SS_SPI-6_ is required for efficient colonization in mice and chickens [47]. As a result, functionality of multiple T6SSs in one strain is diversified rather than redundant in environmental or host adaptability. Remarkably, the gene coding for TOX-REase-5 domain-containing effector was also present in the additional T6SS of strain ATTC 51958. The Imm52 family immunity protein was encoded by the adjacent gene to combat Tox-REase-5 for self-damage protection [48]. Moreover, no SPI-7-like ICE region was found, whose progenitor’s genes in strain CT18 encode a type IV secretion system [49], Vi exopolysaccharide antigen, type IVB pili, and SopE [50]. It is assumed that the genetic loss of these secretion systems may be an attempt by SMEH to reduce the metabolic burden to enhance adaptability.

As widely reported, plasmids and their intermediates serve as the cargo for the acquisition and dissemination of drug resistance genes by conjugation/transposition and site-specific recombination [51], facilitating enhancement of their survival/adaptability [52,53]. Whole-genome sequencing (WGS) or mNGS is an efficient method to rapidly predict AMR genes and has been efficiently applied to epidemiological research [18,54]. Our genomics analysis revealed a consortium of genes responsible for antimicrobial resistance of SMEH. Briefly, *qnrS1* was found in pSMEH1, while *sul2, tet(A), aph(3″)-Ib* and *aph(6)-Id* co-localized onto pSMEH2. Moreover, pSMEH-3 co-harbored *floR* and insertion sites of *tet*(X4). Interestingly, three plasmids identified in this Mbandaka strain presented a core backbone similar to other drug-resistance plasmids, benefiting the accommodation of AMR genes. In accordance with this finding, contextual mobile genetic elements such as insertion sequences (ISs), integrons, and transposons, can effectively capture or disseminate AMR genes. Nevertheless, this observation not only highlights the important role of these elements in the acquisition and spread of antibiotic resistance [55], but also implies the convergence risk of AMR genes and virulence factors driven by diversified mobile elements [56,57].

## 5. Conclusions

To conclude, we isolated a *Salmonella* Mbandaka strain with multidrug resistance from the hydropericardium of a severe patient. We characterized it as the Mbandaka serotype by combining serotyping and MLST, and analyzed its antibiotic susceptibility and virulence potential. We found that the MLST types and AMR patterns of strain SMEH were different from other *Salmonella* strains. Thus, we compared the virulence and resistance genes to assess its infection risk. Sequence analysis revealed that potential transferability of SPIs and other genomic architectures might contribute to its distinct virulence and antibiotic resistance, by comparing typical *Salmonella* isolates. From this scenario, our findings suggest a distinctive evolution process of SMEH compared to other Mbandaka strains, although their phylogenetic clade inference, based on core genomes, is close. Collectively, this study provided further insights into *Salmonella* prevention and treatment, especially for potential AMR spread and clinical therapy.

## Figures and Tables

**Table 1:** Antibiotic susceptibility testing for strain SMEH.

Antimicrobial Type	Antibiotic	Abbr. ^a^	MIC (μg/mL) ^b^	Zone d ^c^ (mm)	R/S/I ^d^
β-lactams	Cefoxitin	FOX	2	-	S
	Cefixime	CFM	<0.25	-	S
	Ampicillin	AMP	<1	26	S
	Amoxycillin/clavulanic acid (augmentin)	AMC	<1/0.5	-	S
	Talampicillin (fisiopen)	TAL	>256	-	R
	Sulbactam and ampicillin	SAM	-	28	S
	Piperacillin	PRL	-	32	S
	Aztreonam	ATM	≤1	32	S
	Ceftazidime	CAZ	-	32	S
	Ceftriaxone sodium	CRO	-	34	S
	Sulbactam and cefoperazone	SCF	-	30	S
	Ticarcillin and clavulanic acid (timetin)	TCC	≤8	-	S
	Piperacillin-tazobactam	TZP	≤4	-	S
	Cefepime	FEP	≤0.12	-	S
	Imipenem	IPM	≤0.25	-	S
	Meropenem	MEM	≤0.25	33	S
Aminoglycosides	Gentamicin	GEN	0.5	20	S
	Streptomycin	STR	>64	-	NB
	Amikacin	AMK	≤2	22	S
	Tobramycin	TOB	≤1	-	S
Quinolones	Ciprofloxacin	CIP	≤0.25	30	I
	Nalidixic acid	NAL	8	-	S
	Levofloxacin	LEV	1	26	I
Phenicols	Chloramphenicol	CHL	>32	6	R
Tetracyclines	Tetracycline	TET	>32	-	R
	Tigecycline	TGC	≤0.5	23	S
	Doxycycline	DOX	≥16	-	R
	Minocycline	MNO	8	-	I
Macrolides	Azithromycin	AZI	8	17	NB
Sulfonamides	Trimethoprim-sulfamethoxazole	SXT	≤20	28	S
Polypeptides	Colistin	COL	0.5	16	NB

^a^ Abbr.: Abbreviation. ^b^: the minimum inhibitory concentrations examined using VITEK 2 Compact AST-N335 with broth micro-dilution. ^c^ d: diameter, determined by the K-B strip on MHA plates. ^d^ R: resistant; S: susceptible, I: intermediate, NB: no clinical breakpoint. Antimicrobial susceptibility testing results were interpreted according to the guidelines recommended by the Clinical and Laboratory Standards Institute (CLSI) with *E. coli* ATCC 25922 as the reference strain. In addition, inhibition zones on Mueller–Hinton agar were measured by Kirby–Bauer disc diffusion assays. Of note, twenty-six and seventeen antimicrobials were applicable to micro-dilution and disk diffusion, whereas “-” denotes no corresponding testing.

## Data Availability

The genome sequences of the SMEH are available in GenBank (chromosome: CP101689, plasmids: CP101690, CP101691, CP101692). The data that support the findings of this study are available from the corresponding author upon reasonable request.

## Supplementary Materials

The following supporting information can be downloaded at: https://www.mdpi.com/article/10.3390/microorganisms12081667/s1, Figure S1 shows colony morphology of strain SMEH cultured on XLD agar medium. Figure S2–S3 shows BLASTp- and COG-based alignment to identify core and specific genes of strain SMEH. Figure S4 shows synteny for T6SSs related to horizontal transfer in the indicated strains. Table S1 shows serotyping results for five representatives of *Salmonella* strains. Table S2 shows general genomic information of *Salmonella* strains SMEH, FORC_015, ATCC 51958, GJ0703, CT18 and 14028S. Table S3 shows the tabular comparison of virulence factors encoded by strains SMEH, FORC_015, ATCC 51958, GJ0703, CT18 and 14028S. Table S4 shows comparative schema amongst the antibiotic resistance determinants predicted in strains SMEH, FORC_015, ATCC 51958, GJ0703 and 14028S.
