# Supplementary material for: Potential Convergence to Accommodate Pathogenicity Determinants and Antibiotic Resistance Revealed in Salmonella Mbandaka
Source: Microorganisms. 2024 Aug 13;12(8):1667. doi: 10.3390/microorganisms12081667 (PMC11357217; doi:10.3390/microorganisms12081667)
Supplement: Supplementary file 1 [file microorganisms-12-01667-s001.zip › microorganisms-3143247-supplementary.pdf]

Supplemental Materials

Table S1. Serotyping for strain SMEH and five *Salmonella* representatives.

Strain	O	H1	H2	Serotype
SMEH	7	z10	e,n,z15	Mbandaka
FORC_015	7	z10	e,n,z15	Mbandaka
ATCC 51958	7	z10	e,n,z15	Mbandaka
GJ0703	7	f,g	-	Rissen
CT18	9	d	-	Typhi
14028S	4	i	1,2	Typhimurium

Table S2. General genomic information of *Salmonella* strains SMEH, FORC_015, ATCC 51958, GJ0703, CT18 and 14028S.

**Strain**		**SMEH**	**FORC_015**	**ATCC 51958**	**GJ0703**	**CT18**	**14028S**
Isolation		hydropericardium	blood	foodborne culture	stool	blood	heart and liver
Serotype		Mbandaka	Typhimurium*	Mbandaka	Rissen	Typhi	Typhimurium
MLST		ST-413	ST-413	ST-3016	ST-469	ST-2	ST-19
Genome (bp)	chromosome	4,837,568	4,759,372	4,905,181	4,930,938	4,809,037	4,870,265
[plasmid name if available]	plasmid	10,047			4,657	218,160 [pHCM1]	93,832
		9,208				106,516 [pHCM2]	
		7,769					
CDS	chromosome	4,534	4,336	4,509	4,519	4,361	4,500
	plasmid	14			5	216	106
		10				122	
		13					
G+C%	chromosome	52.1	52.1	52.2	52.1	52.1	52.2
	plasmid	50.6			52.4	47.6	53.1
		56.0				50.6	
		62.1					
tRNA		86	84	84	86	80	86
rRNA		22	22	22	22	22	22
T3SS		2	2	2	2	2	2
T4SS		-	-	2	-	1	-
T6SS		1	1	2	1	1	1

* Revised as serotype Mbandaka in this study.

Table S3. Comparison of virulence factors in strains SMEH, FORC_015, ATCC 51958, GJ0703, CT18 and 14028S.

Virulence factors	Genes	SMEH	FORC_015	ATCC 51858	GJ0703	CT18	14028S
		CP101689	CP011365	CP019183	CP043509	NC_003198	NC_016856
**Capsule**							
Vi antigen	*tviA*	-	-	-	-	STY4662	-
	tviB	-	-	-	-	STY4661	-
	tviC	-	-	-	-	STY4660	-
	tviD	-	-	-	-	STY4659	-
	tviE	-	-	-	-	STY4656	-
	vexA	-	-	-	-	STY4655	-
	vexB	-	-	-	-	STY4654	-
	vexC	-	-	-	-	STY4653	-
	vexD	-	-	-	-	STY4652	-
	vexE	-	-	-	-	STY4651	-
**Fimbrial adherence determinants**							
Agf/Csg	*csgA*	NOV91_21415	ZV79_1080	SEEM1958_014900	FZX11_14480	STY1181	STM14_1310
	csgB	NOV91_21410	ZV79_1079	SEEM1958_014905	FZX11_14485	STY1180	STM14_1309
	csgC	NOV91_21420	ZV79_1081	SEEM1958_014895	FZX11_14475	STY1182	STM14_1311
	csgD	NOV91_21405	ZV79_1078	SEEM1958_014915	FZX11_14490	STY1179	STM14_1306
	csgE	NOV91_21400	ZV79_1077	SEEM1958_014920	FZX11_14495	STY1178	STM14_1305
	csgF	NOV91_21395	ZV79_1076	SEEM1958_014925	FZX11_14500	STY1177	STM14_1304
	csgG	NOV91_21390	ZV79_1075	SEEM1958_014930	FZX11_14505	STY1176	STM14_1303
Bcf	*bcfA*	NOV91_15965	ZV79_22	SEEM1958_020320	FZX11_19850	STY0024	STM14_0028
	bcfB	NOV91_15970	ZV79_23	SEEM1958_020315	FZX11_19845	STY0025	STM14_0029
	bcfC	NOV91_15975	ZV79_24	SEEM1958_020310	FZX11_19840	STY0026*	STM14_0030
	bcfD	NOV91_15980	ZV79_25	SEEM1958_020305	FZX11_19835	STY0029	STM14_0031
	bcfE	NOV91_15985	ZV79_26	SEEM1958_020300	FZX11_19830	STY0030	STM14_0032
	bcfF	NOV91_15990	ZV79_27	SEEM1958_020295	FZX11_19825	STY0031	STM14_0033
	bcfG	NOV91_15995	ZV79_28	SEEM1958_020290	FZX11_19820	STY0032	STM14_0034
Fim	*fimA*	NOV91_18715	ZV79_556	SEEM1958_017475	FZX11_17070	STY0589	STM14_0635
	fimC	NOV91_18725	ZV79_558	SEEM1958_017465	FZX11_17060	STY0592	STM14_0637
	fimD	NOV91_18730	ZV79_559	SEEM1958_017460	FZX11_17055	STY0593	STM14_0638
	fimF	NOV91_18740	ZV79_561	SEEM1958_017450	FZX11_17045	STY0595	STM14_0640
	fimH	NOV91_18735	ZV79_560	SEEM1958_017455	FZX11_17050	STY0594	STM14_0639
	fimI	NOV91_18720	ZV79_557	SEEM1958_017470	FZX11_17065	STY0590*	STM14_0636
	fimW	NOV91_18760	ZV79_564	SEEM1958_017430	FZX11_17025	STY0600	STM14_0644
	fimY	NOV91_18750	ZV79_563	SEEM1958_017440	FZX11_17035	STY0598	STM14_0642
	fimZ	NOV91_18745	ZV79_562	SEEM1958_017445	FZX11_17040	STY0596	STM14_0641
Lpf	*lpfA*	NOV91_10695	ZV79_3548	-	FZX11_01050	-	STM14_4387
	lpfB	NOV91_10690	ZV79_3547	-	FZX11_01055	-	STM14_4386
	lpfC	NOV91_10685	ZV79_3546	-	FZX11_01060	-	STM14_4385
	lpfD	NOV91_10680	ZV79_3545	-	FZX11_01065	-	-
	lpfE	NOV91_10675	ZV79_3544	-	FZX11_01070	-	STM14_4383
Pef	*pefA*	-	-	-	-	-	-
	pefB	NOV91_14360	ZV79_4178	-	-	-	-
	pefC	-	-	-	-	-	-
	pefD	-	-	-	-	-	-
Peg	*pegA*	-	-	-	-	-	-

	pegB	-	-	-	-	-	-
	pegC	-	-	-	-	-	-
	pegD	-	-	-	-	-	-
Saf	*safA*	-	-	SEEM1958_018685; SEEM1958_018690	-	STY0332	STM14_0352
	safB	-	-	SEEM1958_018680	-	STY0335	STM14_0353
	safC	-	-	-	-	STY0336	STM14_0354
	safD	-	-	-	-	STY0337	STM14_0355
Sef	*sefA*	-	-	-	-	STY4836a*	-
	sefB	-	-	-	-	STY4837	-
	sefC	-	-	-	-	STY4838	-
	sefD	-	-	-	-	STY4839*	-
Sta	*staA*	-	-	SEEM1958_019430	-	STY0207	-
	staB	-	-	SEEM1958_019435	-	STY0206	-
	staC	-	-	SEEM1958_019440	-	STY0205	-
	staD	-	-	SEEM1958_019445	-	STY0204	-
	staE	-	-	SEEM1958_019450	-	STY0203	-
	staF	-	-	SEEM1958_019455	-	STY0202	-
	staG	-	-	SEEM1958_019460	-	STY0201	-
Stb	*stbA*	NOV91_17705	ZV79_354	-	FZX11_18115	STY0373	STM14_0396
	stbB	NOV91_17700	ZV79_353	-	FZX11_18120	STY0372	STM14_0395
	stbC	NOV91_17695	ZV79_352	-	FZX11_18125	STY0371	STM14_0394
	stbD	NOV91_17690	ZV79_351	-	FZX11_18130	STY0370	STM14_0393
	stbE	NOV91_17685	ZV79_350	-	FZX11_18135	STY0369	STM14_0392
Stc	*stcA*	-	-	SEEM1958_008915	FZX11_09040	STY2381	STM14_2655
	stcB	NOV91_03105	ZV79_2095	SEEM1958_008920	FZX11_09045	STY2380	STM14_2654
	stcC	NOV91_03100	ZV79_2094	SEEM1958_008925	FZX11_09050	STY2379	STM14_2653
	stcD	-	-	SEEM1958_008930	FZX11_09055	STY2378	STM14_2652
Std	*stdA*	NOV91_07665	ZV79_2960	SEEM1958_004660	FZX11_04560	STY3177	STM14_3659
	stdB	NOV91_07660	ZV79_2959	SEEM1958_004665	FZX11_04565	STY3176	STM14_3657
	stdC	NOV91_07655	ZV79_2958	SEEM1958_004670	FZX11_04570	STY3175	STM14_3656
Ste	*steA*	NOV91_07250	ZV79_2878	SEEM1958_005105	FZX11_04980	STY3084*	-
	steB	NOV91_07255	ZV79_2879	SEEM1958_005100	FZX11_04975	STY3086	-
	steC	NOV91_07260	ZV79_2880	SEEM1958_005095	FZX11_04970	STY3087	-
	steD	NOV91_07265	ZV79_2881	SEEM1958_005090	FZX11_04965	STY3088	-
	steE	NOV91_07270	ZV79_2882	SEEM1958_005085	FZX11_04960	STY3089	-
	steF	NOV91_07275	ZV79_2883	SEEM1958_005080	FZX11_04955	STY3090	-
Stf	*stfA*	NOV91_16910	ZV79_209	SEEM1958_019360	FZX11_18890	-	STM14_0234
	stfC	NOV91_16915	ZV79_210	SEEM1958_019355	FZX11_18885	-	STM14_0235
	stfD	NOV91_16920	ZV79_211	SEEM1958_019350	FZX11_18880	-	STM14_0236
	stfE	NOV91_16925	ZV79_212	SEEM1958_019345	FZX11_18875	-	STM14_0237
	stfF	NOV91_16930	ZV79_213	SEEM1958_019340	FZX11_18870	-	STM14_0238
	stfG	NOV91_16935	ZV79_214	SEEM1958_019335	FZX11_18865	-	STM14_0239
Stg	*stgA*	-	-	-	-	STY3918	-
	stgB	-	-	-	-	STY3919	-
	stgC	-	-	-	-	STY3920*	-
	stgD	-	-	-	-	STY3922	-
Sth	*sthA*	NOV91_15835	ZV79_4463	SEEM1958_020450	FZX11_19980	STY4944	STM14_5516
	sthB	NOV91_15830	ZV79_4462	SEEM1958_020455	FZX11_19985	STY4943	STM14_5515

	sthC	NOV91_15825	ZV79_4461	SEEM1958_020460	FZX11_19990	STY4941*	STM14_5514
	sthD	NOV91_15820	ZV79_4460	SEEM1958_020465	FZX11_19995	STY4940	STM14_5513
	sthE	NOV91_15815	ZV79_4459	SEEM1958_020470	FZX11_20000	STY4938*	STM14_5512
Sti	*stiA*	NOV91_16785	ZV79_183	SEEM1958_019495	FZX11_19015	-	STM14_0209
	stiB	NOV91_16780	ZV79_182	SEEM1958_019500	FZX11_19020	-	STM14_0208
	stiC	NOV91_16775	ZV79_181	SEEM1958_019505	FZX11_19025	-	STM14_0207
	stiH	NOV91_16770	ZV79_180	SEEM1958_019510	FZX11_19030	-	STM14_0206
Stj	Undetermined	NOV91_15715	ZV79_4438	-	FZX11_20100	-	STM14_5491
	Undetermined	NOV91_15730	ZV79_4441	-	FZX11_20085	-	STM14_5494
	Undetermined	NOV91_15735	ZV79_4442	-	FZX11_20080	-	STM14_5495
	stjB	-	ZV79_4439	-	FZX11_20095	-	STM14_5492
	stjC	NOV91_15725	ZV79_4440	-	FZX11_20090	-	STM14_5493
Stk	*stkA*	NOV91_16830	ZV79_192	-	FZX11_18970	-	-
	stkB	NOV91_16825	ZV79_191	-	FZX11_18975	-	-
	stkC	NOV91_16820	ZV79_190	-	FZX11_18980	-	-
	stkD	NOV91_16815	ZV79_189	-	FZX11_18985	-	-
	stkE	NOV91_16810	ZV79_188	-	FZX11_18990	-	-
	stkF	NOV91_16805	ZV79_187	-	FZX11_18995	-	-
	stkG	NOV91_16800	ZV79_186	-	FZX11_19000	-	-
Tcf	*tcfA*	NOV91_17550	ZV79_323	-	FZX11_18325	STY0345	-
	tcfB	NOV91_17555	ZV79_324	-	FZX11_18320	STY0346	-
	tcfC	NOV91_17560	ZV79_325	-	FZX11_18315	STY0347	-
	tcfD	NOV91_17565	ZV79_326	-	FZX11_18310	STY0348	-
**Macrophage inducible genes**							
Mig-14	*mig-14*	NOV91_06385	ZV79_2710	SEEM1958_006040	FZX11_05900	STY2900	STM14_3354
Mig-5	*mig-5*	-	-	-	-	-	-
**Magnesium uptake**							
Mg^{2+} transport	*mgtB*	NOV91_11360	ZV79_3665	SEEM1958_000410	FZX11_00375	STY4023	STM14_4536
	mgtC	NOV91_11365	ZV79_3667	SEEM1958_000405	FZX11_00370	STY4022	STM14_4538
**Nonfimbrial adherence determinants**							
MisL	*misL*	NOV91_11325	ZV79_3659	SEEM1958_000435	FZX11_00435	STY4030*	STM14_4527
RatB	*ratB*	-	-	SEEM1958_007030	-	STY2758*	
ShdA	*shdA*	NOV91_03865; NOV91_05560	ZV79_2512	-	FZX11_06920	STY2755*	STM14_3078
SinH	*sinH*	NOV91_05580	ZV79_2516	SEEM1958_007015	FZX11_06900	STY2762*	STM14_3085
**Regulation**							
PhoPQ	*phoP*	NOV91_21850	ZV79_1168	SEEM1958_014445	FZX11_14045	STY1271	STM14_1409
	phoQ	NOV91_21845	ZV79_1167	SEEM1958_014450	FZX11_14050	STY1270	STM14_1408
**Secretion system**							
T3SS (SPI-1)	*hilA*	NOV91_06865	ZV79_2803	SEEM1958_005530	FZX11_05400	STY2999	STM14_3475
	hilC	NOV91_06820	ZV79_2794	SEEM1958_005575	FZX11_05445	STY2988	STM14_3465
	hilD	NOV91_06860	ZV79_2802	SEEM1958_005535	FZX11_05405	STY2996	STM14_3474
	iacP	NOV91_06890	ZV79_2808	SEEM1958_005505	FZX11_05375	STY3004	STM14_3480
	iagB	NOV91_06870	ZV79_2804	SEEM1958_005525	FZX11_05395	STY3000	STM14_3476
	invA	NOV91_06965	ZV79_2823	SEEM1958_005430	FZX11_05300	STY3019	STM14_3495
	invB	NOV91_06960	ZV79_2822	SEEM1958_005435	FZX11_05305	STY3018	STM14_3494
	invC	NOV91_06955	ZV79_2821	SEEM1958_005440	FZX11_05310	STY3017	STM14_3493

	invE	NOV91_06970	ZV79_2824	SEEM1958_005425	FZX11_05295	STY3020	STM14_3496
	invF	NOV91_06980	ZV79_2826	SEEM1958_005415	FZX11_05285	STY3022	STM14_3498
	invG	NOV91_06975	ZV79_2825	SEEM1958_005420	FZX11_05290	STY3021	STM14_3497
	invH	NOV91_06985	ZV79_2827	SEEM1958_005410	FZX11_05280	STY3023	STM14_3499
	invI	NOV91_06950	ZV79_2820	SEEM1958_005445	FZX11_05315	STY3016	STM14_3492
	invJ	NOV91_06945	ZV79_2819	SEEM1958_005450	FZX11_05320	STY3015	STM14_3491
	orgA	NOV91_06835	ZV79_2797	SEEM1958_005560	FZX11_05430	STY2991	STM14_3469
	orgB	NOV91_06830	ZV79_2796	SEEM1958_005565	FZX11_05435	STY2990	STM14_3468
	orgC	NOV91_06825	ZV79_2795	SEEM1958_005570	FZX11_05440	STY2989	STM14_3467
	prgH	NOV91_06855	ZV79_2801	SEEM1958_005540	FZX11_05410	STY2995	STM14_3473
	prgI	NOV91_06850	ZV79_2800	SEEM1958_005545	FZX11_05415	STY2994	STM14_3472
	prgJ	NOV91_06845	ZV79_2799	SEEM1958_005550	FZX11_05420	STY2993	STM14_3471
	prgK	NOV91_06840	ZV79_2798	SEEM1958_005555	FZX11_05425	STY2992	STM14_3470
	sicA	NOV91_06915	ZV79_2813	SEEM1958_005480	FZX11_05350	STY3009	STM14_3485
	sicP	NOV91_06880	ZV79_2806	SEEM1958_005515	FZX11_05385	STY3002	STM14_3478
	sipD	NOV91_06900	ZV79_2810	SEEM1958_005495	FZX11_05365	STY3006	STM14_3482
	spaO	NOV91_06940	ZV79_2818	SEEM1958_005455	FZX11_05325	STY3014	STM14_3490
	spaP	NOV91_06935	ZV79_2817	SEEM1958_005460	FZX11_05330	STY3013	STM14_3489
	spaQ	NOV91_06930	ZV79_2816	SEEM1958_005465	FZX11_05335	STY3012	STM14_3488
	spaR	NOV91_06925	ZV79_2815	SEEM1958_005470	FZX11_05340	STY3011	STM14_3487
	spaS	NOV91_06920	ZV79_2814	SEEM1958_005475	FZX11_05345	STY3010	STM14_3486
	sprB	NOV91_06815	ZV79_2793	SEEM1958_005585	FZX11_05450	STY2987	STM14_3463
T3SS (SPI-2)	*ssaC*	NOV91_22675	ZV79_1330	SEEM1958_013575	FZX11_13200	STY1726	STM14_1689
	ssaD	NOV91_22680	ZV79_1331	SEEM1958_013570	FZX11_13195	STY1725	STM14_1690
	ssaE	NOV91_22685	ZV79_1332	SEEM1958_013565	FZX11_13190	STY1724	STM14_1691
	ssaG	NOV91_22735	ZV79_1342	SEEM1958_013505	FZX11_13140	STY1714	STM14_1702
	ssaH	NOV91_22740	ZV79_1343	SEEM1958_013500	FZX11_13135	STY1713	STM14_1703
	ssaI	NOV91_22745	ZV79_1344	SEEM1958_013495	FZX11_13130	STY1712	STM14_1704
	ssaJ	NOV91_22750	ZV79_1345	SEEM1958_013490	FZX11_13125	STY1711	STM14_1705
	ssaK	NOV91_22760	ZV79_1347	SEEM1958_013480	FZX11_13115	STY1709	STM14_1707
	ssaL	NOV91_22765	ZV79_1348	SEEM1958_013475	FZX11_13110	STY1708	STM14_1708
	ssaM	NOV91_22770	ZV79_1349	SEEM1958_013470	FZX11_13105	STY1707	STM14_1709
	ssaN	NOV91_22780	ZV79_1351	SEEM1958_013460	FZX11_13095	STY1705	STM14_1711
	ssaO	NOV91_22785	ZV79_1352	SEEM1958_013455	FZX11_13090	STY1704	STM14_1712
	ssaP	NOV91_22790	ZV79_1353	SEEM1958_013450	FZX11_13085	STY1703	STM14_1713
	ssaQ	NOV91_22795	ZV79_1354	SEEM1958_013445	FZX11_13080	STY1702	STM14_1714
	ssaR	NOV91_22800	ZV79_1355	SEEM1958_013440	FZX11_13075	STY1701	STM14_1715
	ssaS	NOV91_22805	ZV79_1356	SEEM1958_013435	FZX11_13070	STY1700	STM14_1716
	ssaT	NOV91_22810	ZV79_1357	SEEM1958_013430	FZX11_13065	STY1699	STM14_1717
	ssaU	NOV91_22815	ZV79_1358	SEEM1958_013425	FZX11_13060	STY1698	STM14_1718
	ssaV	NOV91_22775	ZV79_1350	SEEM1958_013465	FZX11_13100	STY1706	STM14_1710
	sscA	NOV91_22700	ZV79_1335	SEEM1958_013550	FZX11_13175	STY1721	STM14_1695
	sscB	NOV91_22720	ZV79_1339	SEEM1958_013530	FZX11_13155	STY1717	STM14_1699
	sseA	NOV91_22690	ZV79_1333	SEEM1958_013560	FZX11_13185	STY1723	STM14_1693
	sseB	NOV91_22695	ZV79_1334	SEEM1958_013555	FZX11_13180	STY1722	STM14_1694
	sseC	NOV91_22705	ZV79_1336	SEEM1958_013545	FZX11_13170	STY1720	STM14_1696
	sseD	NOV91_22710	ZV79_1337	SEEM1958_013540	FZX11_13165	STY1719	STM14_1697
	sseE	NOV91_22715	ZV79_1338	SEEM1958_013535	FZX11_13160	STY1718	STM14_1698
	ssrA	NOV91_22665	ZV79_1328	SEEM1958_013585	FZX11_13210	STY1728	STM14_1687

	ssrB	NOV91_22660	ZV79_1326	SEEM1958_013590	FZX11_13215	STY1729	STM14_1686
T3SS effectors translocated via both systems	*slrP*	NOV91_20150	ZV79_833	SEEM1958_016200	FZX11_15815	STY0833*	STM14_928
	sspH1	-	-	-	-	-	-
T3SS-1 translocated effectors	*avrA*	NOV91_06810	ZV79_2792	SEEM1958_005590	FZX11_05455	-	STM14_3462
	sipA	NOV91_06895	ZV79_2809	SEEM1958_005500	FZX11_05370	STY3005	STM14_3481
	sipB	NOV91_06910	ZV79_2812	SEEM1958_005485	FZX11_05355	STY3008	STM14_3484
	sipC	NOV91_06905	ZV79_2811	SEEM1958_005490	FZX11_05360	STY3007	STM14_3483
	sopA	NOV91_02675	-	SEEM1958_009385	FZX11_09530	STY2275*	STM14_2557
	sopB/sigD	NOV91_21155	ZV79_1026	SEEM1958_015200	FZX11_14750	STY1121	STM14_1237
	sopD	NOV91_07200	ZV79_2866	SEEM1958_005170	FZX11_05035	STY3073	STM14_3550
	sopE2	NOV91_01550	ZV79_1802	SEEM1958_010955	FZX11_10675	-	STM14_2244
	sopE	-	-	-	-	STY4609	-
	sptP	NOV91_06875	ZV79_2805	SEEM1958_005520	FZX11_05390	STY3001	STM14_3477
T3SS-2 translocated effectors	*gogB*	-	-	-	-	-	STM14_3164
	pipB2	NOV91_06375	ZV79_2708	SEEM1958_006055	FZX11_05915	STY2897	STM14_3350
	pipB	NOV91_21140	ZV79_1024	SEEM1958_015215	FZX11_14760	STY1117	STM14_1233
	sifA	NOV91_21815	-	SEEM1958_014485	FZX11_14080	STY1264	STM14_1400
	sifB	NOV91_00240	ZV79_1546	SEEM1958_012360	FZX11_12105	STY1462	STM14_1940
	sopD2	-	-	-	-	STY0971*	-
	spiC/ssaB	NOV91_22670	ZV79_1329	SEEM1958_013580	FZX11_13205	STY1727	STM14_1688
	spvC	-	-	-	-	-	-
	spvD	-	-	-	-	-	-
	sseF	NOV91_22725	ZV79_1340	SEEM1958_013515	FZX11_13150	STY1716	STM14_1700
	sseG	NOV91_22730	ZV79_1341	SEEM1958_013510	FZX11_13145	STY1715	STM14_1701
	sseI/srfH	-	-	-	-	-	STM14_1193
	sseJ	NOV91_00385	ZV79_1576	SEEM1958_012195	FZX11_11940	STY1439a*	STM14_1974
	sseK1	NOV91_13480	ZV79_4020	SEEM1958_023030	FZX11_22605	-	STM14_4996
	sseK2	NOV91_03035	-	SEEM1958_008990	FZX11_09120	-	STM14_2428
	sseL	NOV91_03720	ZV79_2217	SEEM1958_008265	FZX11_08435	STY2517	STM14_2824
	sspH2	NOV91_01625	ZV79_1817	-	FZX11_10590	STY2467	STM14_1483; STM14_2769
ACE T6SS (*Escherichia*)	*aec15*	-	-	SEEM1958_022205	-	-	-
	aec16	-	-	SEEM1958_022200	-	-	-
	aec17	-	-	SEEM1958_022195	-	-	-
	aec18	-	-	SEEM1958_022190	-	STY1886	-
	aec19	-	-	SEEM1958_022185	-	STY1890	-
	aec22	-	-	SEEM1958_022175	-	STY1891	-
	aec23	-	-	SEEM1958_022170	-	-	-
	aec24	-	-	SEEM1958_022165	-	-	-
	aec25	-	-	SEEM1958_022160	-	-	-
	aec26	-	-	SEEM1958_022155	-	-	-
	aec27/clpV	-	-	SEEM1958_022150	-	-	-
	aec28	-	-	SEEM1958_022145	-	-	-
	aec29	-	-	SEEM1958_022140	-	-	-
	aec30	-	-	SEEM1958_022135	-	-	-
	aec32	-	-	SEEM1958_022125	-	-	-
**Serum resistance**							
Rck	rck	-	-	-	-	-	-
**Stress adaptation**							
SodCI	*sodCI*	-	-	-	-	-	STM14_1184

**Toxin**							
SpvB	*spvB*	-	-	-	-	-	-
Typhoid toxin	*cdtB*	-	-	SEEM1958_014375	-	STY1886	-
	pltA	-	-	SEEM1958_014390	-	STY1890	-
	pltB	-	-	SEEM1958_014395	-	STY1891	-
**Adherence**							
K88 fimbriae (*Escherichia*)	*faeC*	NOV91_14365	ZV79_4179	-	-	-	-
	faeD	NOV91_14370	ZV79_4180	-	-	-	-
	faeE	NOV91_14375	ZV79_4181	-	-	-	-
	faeE	NOV91_14380	ZV79_4182	-	-	-	-
	faeH	NOV91_14390	ZV79_4184	-	-	-	-
	faeI	NOV91_14395	ZV79_4185	-	-	-	-
**Invasion**							
Invasin A (Yersinia)	-	-	-	SEEM1958_000165; SEEM1958_001785; SEEM1958_002585; SEEM1958_004820; SEEM1958_012075; SEEM1958_013180; SEEM1958_016625; SEEM1958_021080; SEEM1958_021735; SEEM1958_023070; SEEM1958_023585; SEEM1958_023620	-	-	STM14_0079; STM14_1065; STM14_2031; STM14_2198; STM14_2376; STM14_3030; STM14_3980; STM14_4190; STM14_936
Invasion of brain endothelial cells (Ibes) (*Escherichia*)	*ibeB*	-	-	-	FZX11_01585	-	-
**Immune evasion**							
LPS glucosylation (*Shigella*)	*gtrA*	NOV91_04275; NOV91_05305	-	-	FZX11_07880	-	-
**Anaerobic respiration**							
Fused nitrate reductase (Mycobacterium)	*narX*	-	ZV79_1711	-	-	-	-
**Others**							
O-antigen (Yersinia)		NOV91_02780	ZV79_2029	SEEM1958_009285	FZX11_09430	-	-

Table S4. Comparison of genes accounting for antimicrobial resistance in strains SMEH, FORC_015, ATCC 51958, GJ0703 and 14028S.

Resistance	Gene	SMEH	FORC_015	ATCC 51958	GJ0703	14028S
Tetracycline	*tet(A)*	NOV91_23640 [pSMEH2]	-	-	FZX11_01400	-
Sulfonamide	*sul3*	-	-	-	FZX11_01495	-
	sul2	NOV91_23615 [pSMEH2]	-	-	-	-
	sul1	-	-	-	FZX11_01470	-
Quinolone	*qnrS1*	NOV91_23565 [pSMEH1]	-	-	-	-
Disinfectant	*qacE*	-	-	-	FZX11_01465	-
Phenicol	*floR*	NOV91_23675 [pSMEH3]	-	-	-	-
Trimethoprim	*dfrA12*	-	-	-	FZX11_01450	-
β-lactam	$bla_{TEM\text{-}1B}$	-	-	-	FZX11_01525	-
Aminoglycoside	*aph(6)-Id*	NOV91_23625 [pSMEH2]	-	-	-	-
	aph(3'')-Ib	NOV91_23620 [pSMEH2]	-	-	-	-
	aadA2	-	-	-	FZX11_01460	-
	aadA1	-	-	-	FZX11_01515	-
	aac(6')-Iaa	NOV91_00325	ZV79_1563	SEEM1958_012275	FZX11_12020	STM14_1958

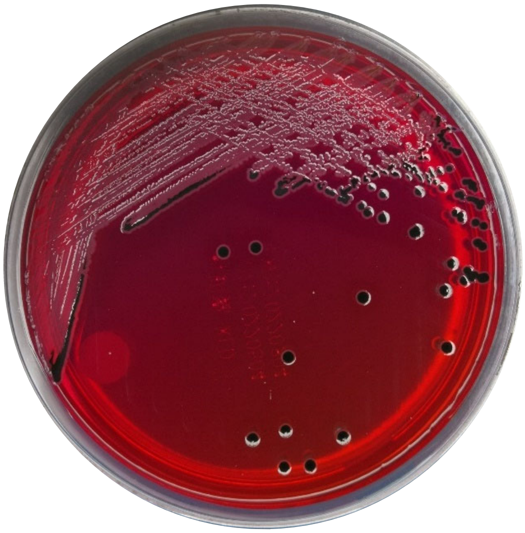

Figure S1. Colony morphology of strain SMEH cultured on XLD agar medium.

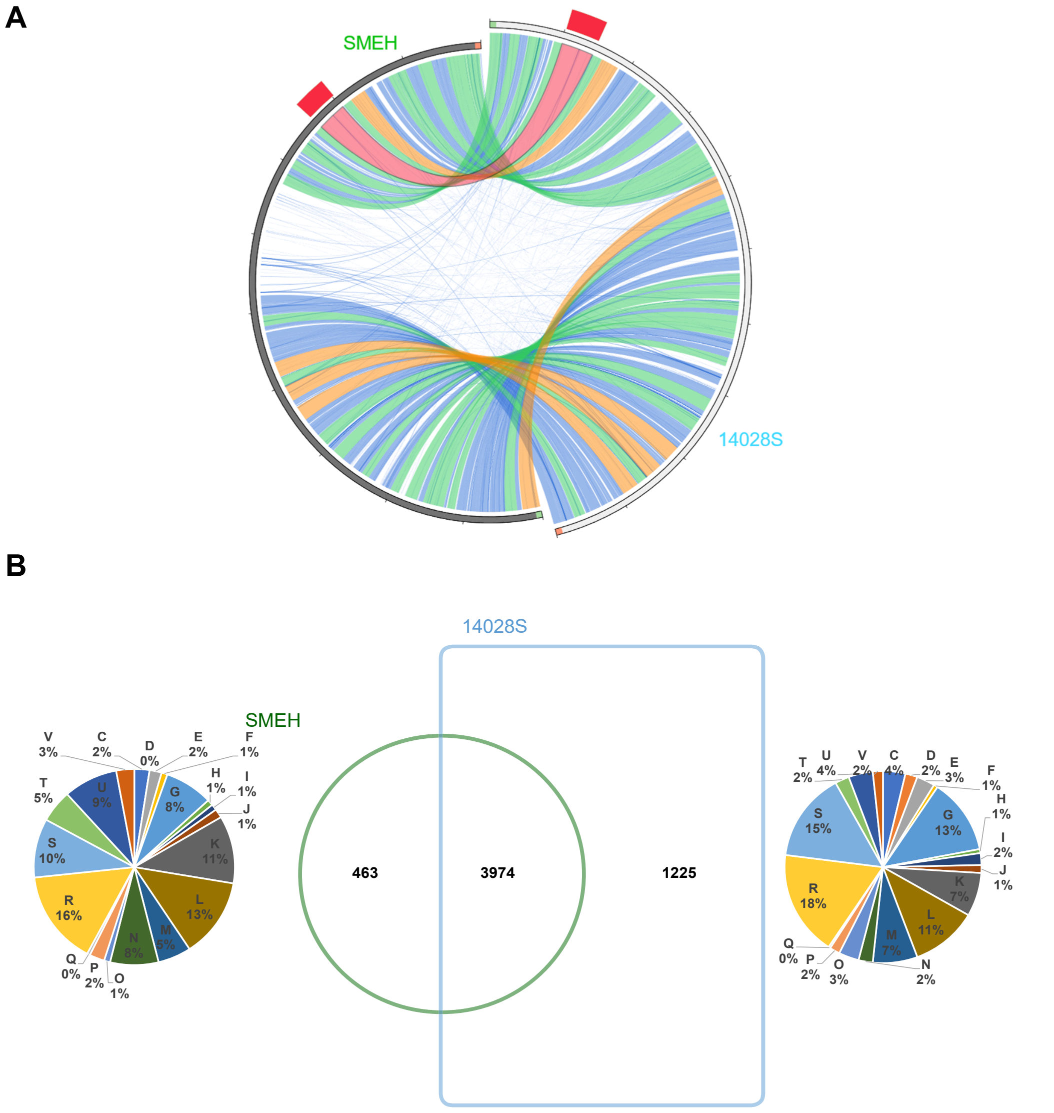

Figure S2. Comparative genomics of *Salmonella* strains 14028S and SMEH. **(A)** Full-length circos-based schematics of *Salmonella* strains 14028S and SMEH. Color intensity of interconnected lines indicates the sequence similarity. **(B)** BLASTp- and COG-based (cluster of orthologous groups) alignment to identify core and specific genes of SMEH chromosomal genes. All-against-all BLASTp was iteratively conducted for this analysis (E-value = 0.01, identity = 30% as thresholds). COG classification was conducted by RPS-BLAST against clusters of orthologous groups (COG) database available at NCBI (ncbi.nlm.nih.gov/COG) with E-value ⩽ 0.01.

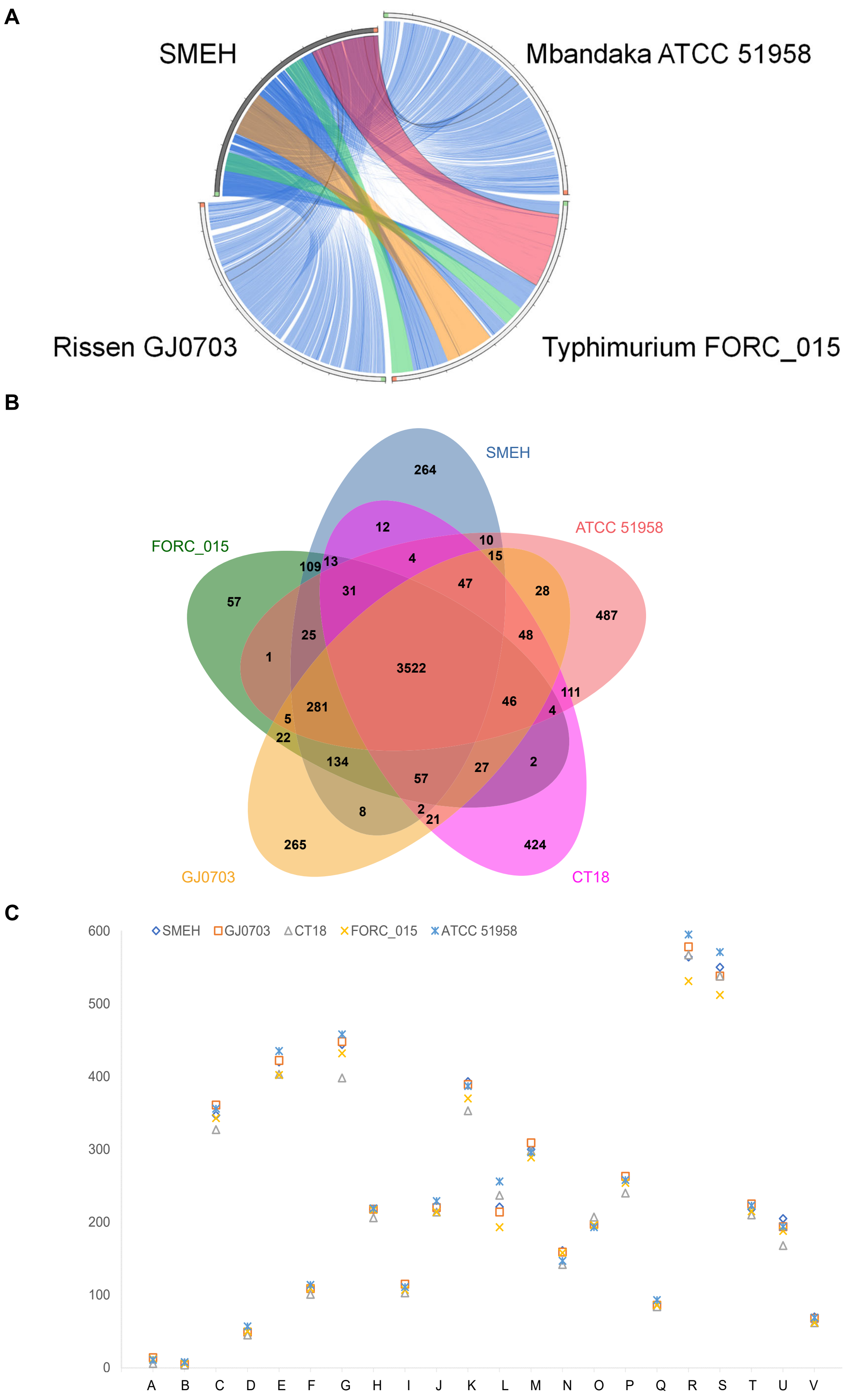

A
SMEH
Mbandaka ATCC 51958
Rissen GJ0703
Typhimurium FORC_015
B
SMEH
ATCC 51958
FORC_015
GJ0703
CT18
264
12
10
15
4
109
13
31
47
28
487
57
25
48
1
3522
111
46
4
281
5
22
134
57
27
2
8
2
21
265
424
C
SMEH
GJ0703
CT18
FORC_015
ATCC 51958
600
500
400
300
200
100
0
A B C D E F G H I J K L M N O P Q R S T U V

Figure S3. Comparative genomics of *Salmonella* strains CT18, GJ0703, FORC_015, ATCC 51958 and SMEH. **(A)** Full-length circos-based schematics of *Salmonella* strains CT18, GJ0703, FORC_015, ATCC 51958 and SMEH. Color intensity of interconnected lines indicates the sequence similarity. **(B)** BLASTp- and **(C)** COG-based (cluster of orthologous groups) alignment to identify core and specific genes of SMEH chromosomal genes. All-against-all BLASTp was iteratively conducted for this analysis (E-value = 0.01, identity = 30% as thresholds). COG classification was conducted by RPS-BLAST against clusters of orthologous groups (COG) database available at NCBI (ncbi.nlm.nih.gov/COG) with E-value $\leqslant$ 0.01.

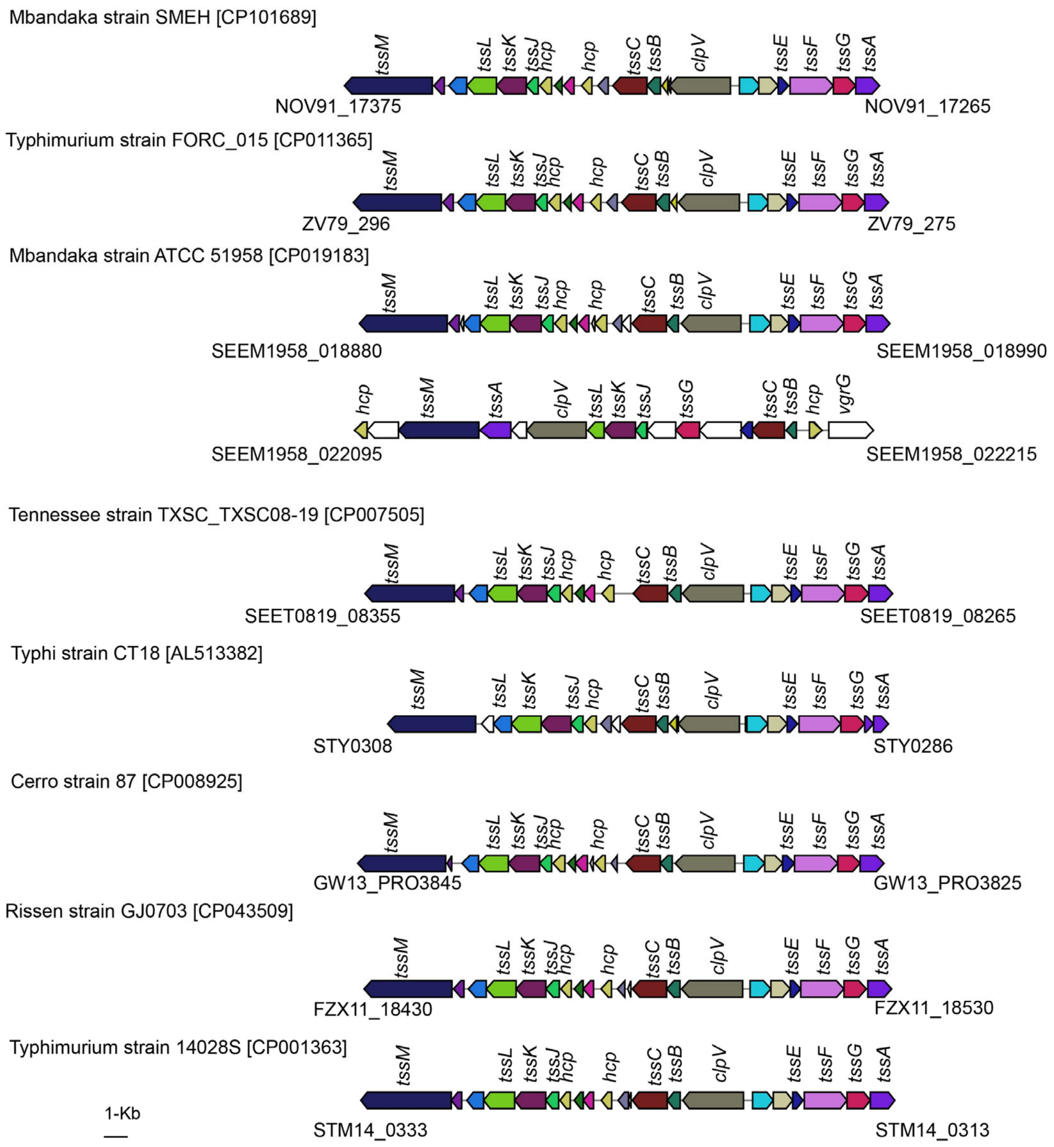

Figure S4. Synteny for T6SSs related to SPIs in *Salmonella* strains under study. Of note, there are two T6SS in strain ATCC 51958. The SPI-6 homologs of SMEH were co-linearly compared with the counterparts in the genomes of strains Typhimurium FORC_015, Mbandaka ATCC 51958, Tennessee TXSC_TXSC08-19, Typhi CT18, Cerro 87 and Rissen GJ0703. The genes of mosaic T6SS homologs identified in a variety of *Salmonella* genomes were color coordinated, whereas specific genes and genomic contexts were indicated by white.
